# Government resource contributions to the private-not-for-profit sector in Uganda: evolution, adaptations and implications for universal health coverage

**DOI:** 10.1186/s12939-018-0843-8

**Published:** 2018-10-05

**Authors:** Aloysius Ssennyonjo, Justine Namakula, Ronald Kasyaba, Sam Orach, Sara Bennett, Freddie Ssengooba

**Affiliations:** 1 Department of Health Policy, Planning and Management, Makerere University School of Public Health, P.O Box 7072, Kampala, Uganda; 2 Uganda Catholic Medical Bureau, Uganda Catholic Secretariat, Nsambya Hill, 672 Hanlon Road, P. O. Box 2886, Kampala, Uganda; 3 Department of International Health, Johns Hopkins Bloomberg School of Public Health, 615 North Wolfe Street, Baltimore, MD 21205 USA

**Keywords:** Government subsidies, Primary health care, Private-not-for-profit, Non-state providers, Universal health coverage, Uganda, Complex adaptive systems

## Abstract

**Background:**

A case study was prepared examining government resource contributions (GRCs) to private-not-for-profit (PNFP) providers in Uganda. It focuses on Primary Health Care (PHC) grants to the largest non-profit provider network, the Uganda Catholic Medical Bureau (UCMB), from 1997 to 2015. The framework of complex adaptive systems was used to explain changes in resource contributions and the relationship between the Government and UCMB.

**Methods:**

Documents and key informant interviews with the important actors provided the main sources of qualitative data. Trends for GRCs and service outputs for the study period were constructed from existing databases used to monitor service inputs and outputs. The case study’s findings were validated during two meetings with a broad set of stakeholders.

**Results:**

Three major phases were identified in the evolution of GRCs and the relationship between the Government and UCMB: 1) Initiation, 2) Rapid increase in GRCs, and 3) Declining GRCs. The main factors affecting the relationship’s evolution were: 1) Financial deficits at PNFP facilities, 2) advocacy by PNFP network leaders, 3) changes in the government financial resource envelope, 4) variations in the “good will” of government actors, and 5) changes in donor funding modalities. Responses to the above dynamics included changes in user fees, operational costs of PNFPs, and government expectations of UCMB. Quantitative findings showed a progressive increase in service outputs despite the declining value of GRCs during the study period.

**Conclusions:**

GRCs in Uganda have evolved influenced by various factors and the complex interactions between government and PNFPs. The Universal Health Coverage (UHC) agenda should pay attention to these factors and their interactions when shaping how governments work with PNFPs to advance UHC. GRCs could be leveraged to mitigate the financial burden on communities served by PNFPs. Governments seeking to advance UHC goals should explore policies to expand GRCs and other modalities to subsidize the operational costs of PNFPs.

## Background

The universal health coverage (UHC) agenda, as framed under Sustainable Development Goal (SDG) 3, seeks to keep health care services affordable while expanding coverage to reach those most in need, as well as increase the quality and diversity of interventions to promote well-being and healthy lifestyles [1, 2]. In countries such as Uganda, these goals have helped to focus the reorganization of health care service delivery systems on meeting the health needs of the population and creating sustainable financing mechanisms [3, 4]. The Ministry of Health (MoH) serves as the steward of the Ugandan health system, working closely with other line ministries, such as the Ministry of Finance, Planning and Economic Development (MoFPED) and local governments. Over the years, Uganda has developed a mixed health system with government and private sectors making contributions to the delivery of health services. Uganda’s public health care system is decentralized. At district level, health care is delivered by community health workers, three types of health centres (HCs), and general hospitals under the stewardship of local governments. Semi-autonomous regional and national referral hospitals provide specialized care [5]. The private sector is diverse, encompassing private not-for-profit (PNFP) providers, private for-profit health providers, and traditional and complementary medical practitioners [6]. The public system—mostly financed by government and donor funds—is the dominant provider of health services in the country, but the PNFP sector has also emerged as a prominent contributor [7, 8].

The Uganda National Minimum Health Care Package (UNMHCP) is the basic package of health care services required to be provided by both the public and private sectors (MoH 2010). The Uganda demographic and health surveys indicate that the private sector overall provides between 60 and 70% of the frontline health services; of this, the PNFP sub-sector provides 42% of the total [9, 10]. In terms of infrastructure, PNFPs operate 40% of hospitals and 22% of lower-level health facilities [11]. According to a situation analysis prepared by the MoH, the workforce in the PNFP sector constitutes nearly a quarter of the total health workforce in the country, about 9000 professionals [12].

Studies have shown that the Ugandan population seeks care in complex ways, moving among PNFPs, government facilities, and other service providers, and that the PNFP sector is trusted and perceived to provide satisfactory quality [13, 14]. Systematically engaging with PNFPs to provide health services has the potential to play a major role as Uganda embarks on an ambitious agenda to move towards UHC.

The main sources of health care financing in Uganda are government revenue, private funds (voluntary prepayment and out-of-pocket expenditures) and donor grants/loans accounting for 15%, 38% and 47% respectively of the total health expenditure in 2012 [15]. Public funds used to pay for health services in public facilities are typically not directly linked to specific outputs or outcomes [15]. Since 1997, in an attempt to increase access to services, the government has also supported the PNFP sector. These government resource contributions (GRCs) include financial and non-financial support. Financial GRCs are primarily Primary Health Care (PHC) grants (that cover operational costs for health centers, hospitals and training schools), as well as medicines and wage subventions. Other resources provided include equipment contributions (such as ambulances and diagnostics) and staff training [10, 16, 17].

The goal of the current study was to document and analyze GRCs to PNFPs in Uganda from 1997 to 2015; it sought to understand the processes, mechanisms and dynamics that explain the evolution of GRCs and to analyze how government-PNFP relationships have adapted over time. This paper addresses two questions: 1) What have been the trends in key service outputs and GRCs to the PNFP sector from 1997 to 2015?, and 2) What explains the evolution of the PHC grants (the predominant form of GRC) and adaptations by both the government and the PNFP sector over the years?

## Theoretical approach

This study utilized complex adaptive systems (CAS) theory to explain how the complex changes that occurred in the broader context and trends in PHC grants elicited adaptations by government and PNFPs [18, 19]. CAS theory asserts that a “systems environment” comprises interrelating agents that can self-organize, adapt and learn from experience [18]. Several studies have effectively used CAS theory to understand complex phenomena such as dual practice, immunization services and neonatal mortality in Uganda [20–22] and the development of rural health systems in China [23]. CAS is suited to understanding complex problems characterized by a multiplicity of interacting agents, changes in the context and shifting patterns of interaction [24].

This study adopted an approach used by Paina and Peters: using causal loop diagrams (CLDs) to illustrate complexity, particularly cause and effect dynamics [20, 25]. According to Paina and Peters, feedback loops are created by complex interactions such that an action by one actor causes anticipated and unanticipated actions by other actors in the contextual environment. CLDs are utilized in this paper to illustrate the evolution of relationships between the government and PNFPs over three distinct phases.

## Methods

This case study examines GRCs (particularly the PHC grants) to the PNFP sector in Uganda between 1997, when PHC grants were initiated, and 2015. It focuses on the case of the Uganda Catholic Medical Bureau (UCMB) network. UCMB was selected because it is the largest PNFP network of facilities and training schools in Uganda; it also has a well-established data archive that enabled the trend analysis of GRCs and service outputs over time. In addition to UCMB, the PNFP sector also includes the Uganda Protestant Medical Bureau (UPMB), the Uganda Orthodox Medical Bureau (UOMB) and the Uganda Muslim Medical Bureau (UMMB) [26].

Quantitative analysis of trends in PHC grant allocations and service outputs were combined with qualitative analysis of key informant interviews (KIIs) and relevant documents to explain systems dynamics and develop CLDs. The case study’s observations and conclusions were validated by experienced current and former actors in both PNFPs and government.

### Interview data

Data were collected in four of Uganda’s 112 districts purposively selected because they contain the highest number of accredited UCMB health facilities: greater Mukono, Kampala, Arua, and greater Gulu. These districts also host regional/diocesan coordination offices for the UCMB. A total of 39 interviews were conducted in 2016 with key informants, including representatives of the central government (MoH and line ministries) and district authorities, representatives from other Medical Bureaus, development partners, and facility managers (see Table 1). Interviewees were purposively selected based on their current or previous roles in government and the national and sub-national health care landscape. Respondents’ roles ranged from managing the disbursement of funds to grant administration, coordination and service delivery. All respondents were asked to provide written consent to participate in the study and for audio recording of interviews. An interview guide developed for the study covered several domains including: explanations for the trends in GRCs, main adaptations made by both government and the PNFPs, effects of the adaptations, and recommendations for future improvements. All interviews were conducted in English, recorded, and then transcribed verbatim.

**Table 1:** KII participants at national and sub-national levels

Level	Category	Institutions or offices represented	Number
National/central	Government	MoH, MoFPED, Ministry of Local Government (MoLG), and National Medical Stores (NMS)	8
	PNFP	UCMB Secretariat in Kampala, Diocesean health coordinator in Kampala and Joint Medical Stores (JMS), other PNFP bureaus particularly Uganda Protestant Medical Bureau(UPMB) and Uganda Moslem Medical Bureau(UMMB)	8
	Development partners	World Bank, Doctors with Africa (CUAMM), Association of Volunteers in International Service (AVSI) Foundation	6
Sub-national/ district	Government	District Health Officers (DHOs), Chief Administrative Officers (CAOs), Local Council (District) councilors	5
	PNFP	Diocesan Health Coordinators	3
		Facility Managers	9
Total interviews			39

### Document review

A literature search conducted for resources related to the relationships between the government and PNFPs, particularly with respect to the PHC grants, resulted in 36 documents. Most of these came from the UCMB archives, supplemented with additional documents recommended by key informants. Each selected document was reviewed using a matrix that included: document description (e.g., title, source, language, publication status, intervention context), scope (national or sub-national), and thematic focus (evolution of GRCs, players, processes, dynamics and mechanisms, and adaptations within PNFPs and government).

### Secondary quantitative data

Secondary data were extracted from UCMB databases on the following service delivery outputs: out-patient department attendance, admissions, in-patient days, deliveries, ante-natal care attendance, and immunization. These data were used to study trends in service delivery outputs between 1997 and 2015. Data on PHC grant allocations was extracted from a comprehensive database on PHC allocations from MoFPED from 1997 to 2015 maintained by UCMB.

### Ethical clearance

Ethical clearance was granted by the Higher Degrees, Research and Ethics Committee of the Makerere University School of Public Health, the Uganda National Council for Science and Technology (UNCST) and the WHO Ethics Review committee.

### Data analysis

Interview transcripts were coded in Atlas.ti (v. 7.0). Query reports (lists of quotations that relate to the given theme) were generated and analyzed using thematic analysis and concepts from CAS theory. The analysis was organized around: context for PHC grants, mechanisms (explanations), outcomes, and how the relationships between PNFPs and government actors evolved over time.

Causal loop diagrams (CLD) were developed using Vensim PLE Plus [27] to illustrate iterative interactions among contextual factors, events, actors, PHC grant disbursements, and adaptations in the relationships between the government and PNFP providers. The CLDs were refined through an iterative process by the authors, and subsequently validated at stakeholder meetings. These processes were complementary and helped generate the most plausible relationships, interactions and feedback loops. Standard notation proposed by Vensim (2017) was used in the study. A positive arrow (+) was used where a change in factor X caused factor Y to change in the same direction. A negative arrow (−) was used where a change in factor X caused an opposite change in factor Y. Some interactions cause reinforcing feedback loops in which an increase in X causes more of Y, which in turn leads to more of X. Similarly, negative feedback loops exist where variables influence each other in opposite directions. The thickness of the arrow denotes the researchers’ estimation of the relative significance of the relationship.

Quantitative data from UCMB databases were exported into Excel for analysis. For PHC grant disbursements, the absolute amounts were adjusted for inflation using 2010 as the base year.

## Results

### Trends in government grants to PNFPs and selected service outputs

**Fig. 1:**
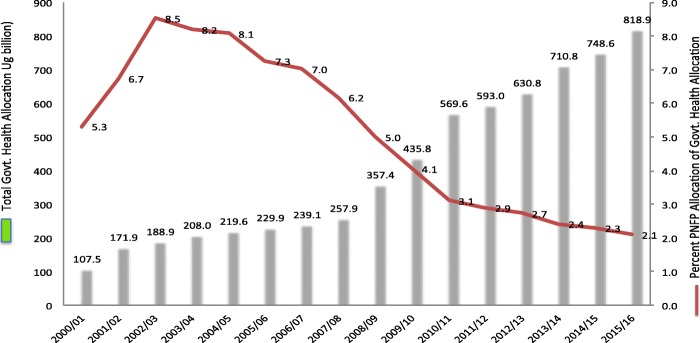
Trend for Total Govt. Health budget and percent share of government resources going to PNFPs over the years

Figure 1 depicts the percentage share of allocation to PNFPs as a proportion of the government health care budget. A decline is observed starting in 2006.

**Fig. 2:**
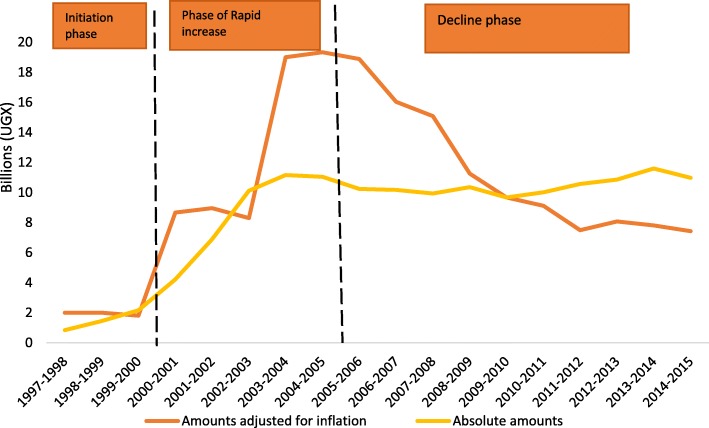
PHC Grant disbursements to UCMB network in absolute terms and values adjusted for inflation

Figure 2 shows trends in PHC grant disbursements to UCMB facilities for the period 1997–2015. In real terms, PHC grant disbursement can be divided into three phases: 1) initiation (1997–2000), 2) increase (2000–2005), and 3) decline (2006–2015). In the three-year initiation phase (between 1997 and 2000), PHC grants ranged from 1.8 to 2.0 billion Uganda shillings each year in real terms (1USD = 2177.56 Ugandan Shillings, with 2010 as base year). This was followed by a sharp increase from 8.67 billion to 19.34 billion shillings between 2001 and 2005. The trends indicate a decline in PHC grants in real terms after adjusting for inflation from 18.88 to 7.43 billion shillings from 2006 to 2015 respectively.

**Fig. 3:**
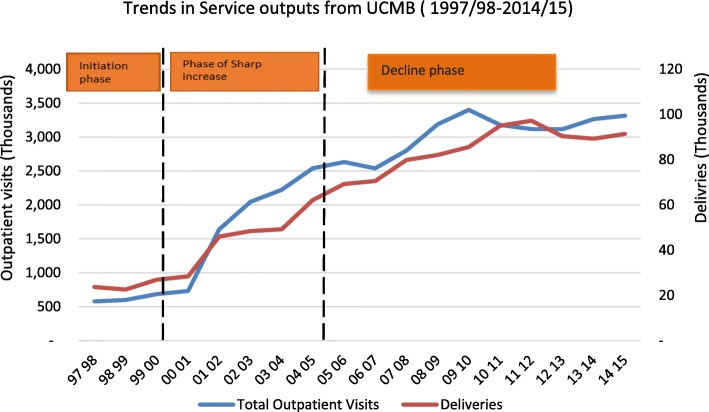
Trends in deliveries and OPD utilization in the UCMB network-1997 0 2015

The increase phase began when allocations for UCMB (and other PNFP providers) expanded during the period from 2000 to 2005 During this time, PHC grant allocations expanded from only financing hospitals to also including lower level health centers and training schools affiliated to PNFPs [28]. As shown in Fig. 3, PHC grants enabled the PNFP sector to contribute to UHC objectives by reducing user fees, increasing service outputs and utilization of services, and increasing the population served. Interviewees confirmed that during this period many facilities provided subsidized services and expanded free services, especially for HIV treatment and immunization. One PNFP respondent said:

*“HIV patients come here in large numbers....When the government funds came in, we were able to give those drugs free or buy drugs using government funds. Now patients are able to come here to access the HIV clinic and other clinics…without pay….It [PHC grant] is subsidizing the bill.”* P10: KII_ National level_ PNFP.

As illustrated in Fig. 3, service outputs, including outpatient visits and maternal deliveries, have gradually increased over the study period. This presents a contrast to the declining real value of PHC grants disbursed since 2005.

### Phase 1: Initiation (1997–2000)

**Fig. 4:**
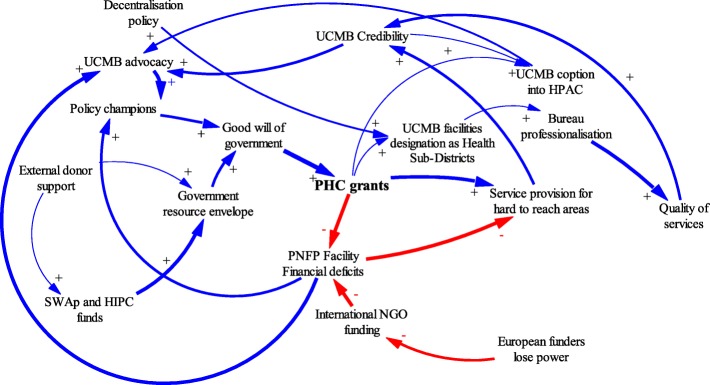
Causal Loop diagram for ‘initiation phase’ of PHC grant

Figure 4 shows the factors that influenced the initiation of PHC grants and the adaptions made by government and PNFP networks during this period.

The key factors that led to the initiation of PHC grants were internal factors among PNFPs and a favorable context for government financing of health services.

Document reviews and narratives from interviews indicated that prior to this phase, the 1990s were characterized by financial distress among PNFPs. Many PNFP facilities had relied heavily on donor and missionary funding from Europe in the 1990s, this external funding to PNFPs *“had stopped,” “dwindled,” or “dried up.”* This was partly due to political transitions in Europe: loss of power by political parties that had supported the provision of foreign aid to missionaries operating in countries like Uganda [29]. Likewise, the end of the civil conflict in Uganda in the 1980s reduced the allocation of foreign aid from European-based missionaries that supported PNFP providers. These reductions in financing threatened the survival of PNFP facilities. In response, hospital managers started actively lobbying and putting pressure on the government to provide financial support in order to “save” PNFP facilities. Interviewees indicated that the attention of the government was increased when advocates used terms like “hard-to-reach,” and “underserved by government” to describe their patient populations, and by threats of hospital closures. In one instance, a participant reported that:

*“the keys to a certain PNFP hospital were brought to the MoH to threaten the closure of a hospital if funding from the government was not provided”*[…] P11: KII_ Former Administrator_National level_ Government.

This coincided with a favorable context for increased government funding. The government resource envelope was increasing as a result of the adoption of a Sector-Wide Approach (SWAp) to financing government services; further, debt relief for Highly Indebted Poor Countries (HIPC) was conditioned on financing health and other development sectors [30]. Under SWAp [8, 17] all stakeholders, including government, donors and the private sector, were encouraged to identify priority sector programs, such as in the area of health, and to pool resources to address problems.

SWAp also created avenues for PNFP providers to participate formally in policy development processes, such as sector advisory committees. PNFP providers used these government structures to lobby for funding. The financial challenges identified within the PNFP sector were high on the policy agenda for MoH and SWAp in the initiation phase.

Champions for health in the government reportedly enabled the MoH to initiate support to PNFP providers. Discussions at the validation meetings for this study indicated that Dr. Chrispus Kiyonga, the Minister of Health at the time and a strong ally of PNFP providers, had previously served as the Minister of Finance and thus, well informed about government-wide revenues, funding horizon and the feasibility of providing subsidies to the PNFP sub sector.

Key policy documents from the time—such as the government decentralization policy, the National Health Policy I (1999–2009) [31] and the first health sector strategic plan from 2000 [32]—both brought to the fore and demonstrated policy makers’ understanding that the government was not able to reach all areas due to limited resources. Particularly in remote rural areas, partnering with non-government providers was essential to providing health care services.

The government thus delegated some health care delivery to PNFP providers in the broader move towards decentralization of services [33, 34]. The government introduced guidelines for PHC grants that stipulated what was expected from PNFPs; these included reducing user fees, expanding geographical coverage and increasing the range of health care services offered [34]. PNFPs were also required to comply with public financing rules and restrictions [34]. Some PNFPs partially resisted these guidelines, perceiving them as an attack on their autonomy. Nevertheless, UCMB and other bureaus organized internally to support their facilities to comply with the guidelines and boost the quality of care offered as means of justifying PHC subsidies from Government. The UCMB network revised its mission in 1999 to signal renewed commitment to professionalization [35].

### Phase II: Increase in PHC grants (2001–2005)

**Fig. 5:**
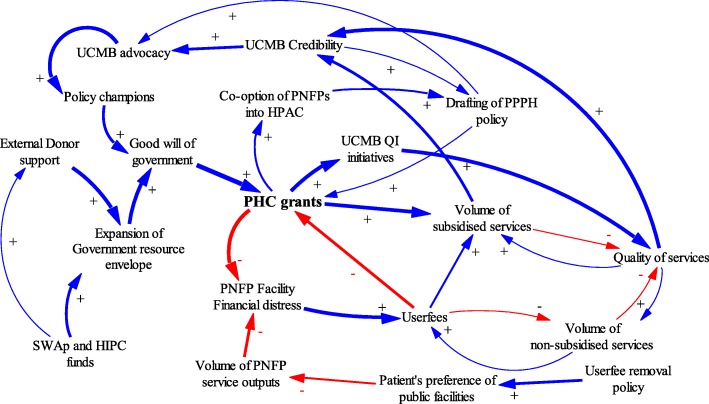
Causal Loop diagram for phase of ‘marked increase’ of in PHC grants and the adaptions by government and UCMB

The period from 2001 to 2005 was characterized by a marked increase in health funding from the government. The health budget as a percentage of the overall government budget increased from 6% in 1999 to 10% in 2004 [36]. Figure 5 illustrates dynamics during this phase, in which PHC grants were significantly increased.

The increase in available resources was enabled by HIPC debt relief, SWAps and new funding from Global Health Initiatives (GHIs). The HIPC debt relief program increased government revenue by reducing loan repayment. GHIs, such as the Global Fund to Fight AIDS, TB and Malaria and the U.S. President’s Emergency Plan for AIDS Relief (PEPFAR), began in this period and dramatically increased resources available to the government for key disease-specific health programs [37, 38].

Increasing government resources enabled several reforms. New policies included designating PNFP facilities as sub-district health headquarters and referral points [39]. Poverty eradication programs, such as the Poverty Eradication Action Plan (PEAP), were started [36–39]. As one interviewee described, health programs were seen as integral to poverty eradication:

*“When Uganda’s debt was forgiven…the country preferred [to use] the money for poverty eradication [programs]. Making PHC grants ‘conditional’ made it look like the funds were really targeting the poor.”* P26: KII_ Former Administrator_ National level_.

The abolition of user fees in public health facilities was considered a major government reform; it featured as a plank in political platforms during the ongoing transition to a multi-party system of government [7, 40]. Some respondents attributed the increase in government budget allocations to the need to compensate both public health facilities and PNFPs for the income lost from rescinding user fees. Other drivers of the funding increases included continued advocacy by PNFPs in health policy fora such as the Health Policy Advisory Committee (HPAC) [17, 41] and continued support from sector leadership.

The reforms created both opportunities and challenges for the PNFPs. Direct opportunities included increases in PHC grant allocations for PNFPs and new opportunities for advocacy. However, a challenge reportedly arose from the mismatch between government expectations and the resource needs of the PNFP sector.

*“[…]We are giving you money to cater for the poor, [but] when you are charging [user fees], you leave the poor ones out[…].Since we are giving you supplementary funding, you should reduce on your user fees, because they are a burden and affect health seeking behaviors.”* P11: KII_ Former Administrator_ National level_.

During this phase, the guidelines for PHC grants were expanded to emphasize reaching poor and hard-to-reach populations with health care services. The PNFPs responded to user fee abolition in the public sector by reducing their fees. They also conducted costing studies to ascertain the cost of delivering services [42]. The abolition of user fees in public health facilities generated a widespread perception that PNFPs also ought to provide free services while maintaining high quality standards [43]. Removing user fees at government facilities reportedly caused patients to shift from PNFPs to government facilities. This negatively affected service volume and increased PNFPs’ risk of financial deficits. Interview and meeting narratives indicated that this created another new dimension for advocacy: PNFPs asked the government to increase their subsidies as they could not lower fees beyond certain levels and remain functional.

During this time (2001–2005), inadequate and variable quality of health services provided by PNFPs was a major concern for both the MoH and the PNFP bureaus. Many PNFP facilities did not meet the prescribed standards for staffing and service mix. Some PNFP facilities were operating without licenses and tended to hire less-qualified staff than public facilities [41, 44, 45]. These concerns prompted professionalization efforts among the PNFPs. UCMB was a front-runner in building institutional structures to support its network. It created a self-regulation system that included annual accreditation of facilities. The main incentive for compliance was eligibility to receive a PHC grant [46–49].

### Phase III: Decline (2006–2015)

**Fig. 6:**
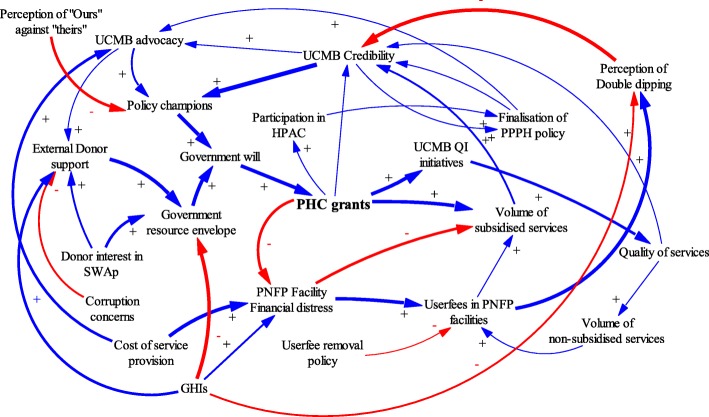
Causal loop diagram for ‘decline phase’ of PHC grants

In the mid-2000s, however, the trend of increasing support to PNFPs reversed. Major explanatory factors were declines in government budgets and changes in leadership at the MoH. Figure 6 illustrates dynamics during the decline phase in PHC grants and general PNFP support.

In the mid-2000s, major leadership changes occurred at the MoH. The new MoH leadership held a different vision for PNFP support. At the local government level, leaders also contested government funding of PNFP providers. These changes coincided with new constraints on the government’s resource envelope due to the abandonment of the SWAp [50–52]. Further, concerns about corruption were prevalent among donors [53–56] at this time. External development partners withdrew funds from the government budget and away from the health sector more broadly, reducing the monies available for PHC grants. Many donors “rationalized” moving their health-related aid away from the government to agencies that were associated with a perception of being relatively less prone to corruption including PNFPs. For instance, the Danish development agency DANIDA stopped funding the government’s National Medical Stores (NMS) and moved all its aid for medicines to the PNFP-owned Joint Medical Stores (JMS). The United States Agency for International Development (USAID) similarly shifted to funding the JMS instead of the NMS. The stakeholders that attended the validation meetings reported that these actions reflected a “divorce” by donors away from government budget contributions.

The growth of GHIs continued during this phase, leading to donor subsidization of HIV services, vaccinations, and malaria interventions. These initiatives benefitted both government and PNFP facilities, but they reduced the pressure on the government to provide direct support to PNFPs using PHC grants [57]. GHIs also started working directly with the PNFP sector, bypassing government. The UCMB became one of the fund-holders for PEPFAR programs in Uganda [10, 16, 58]. GHI funding streams increased pressure on PNFPs to provide free services for HIV, TB and malaria clients but failed to fully appreciate the costs incurred by the PNFP sector [13, 46]. PNFP providers were perceived to have continued recieving financial support from charitable sources, especially from abroad.

Lack of transparency regarding the costs to PNFP providers of service provision and the volume of donations contributed to unfavorable perceptions of PNFP facilities. The PNFP sector was accused of “multiple dipping,” that is, getting money from three sources—government, patients and donors—for each service. This was perceived as unfair, especially by public facilities that were neither charging user fees nor getting charitable donations [10, 16, 59, 60].

Some respondents echoed a common argument that the government should shift its financing to public facilities, which are burdened by increased demand for quality health services and yet receive less money compared to the PNFPs:

*“There was a perception that the PNFPs had more money than the government and so people failed to understand why the government [had to] keep giving money to PNFP…They literally ignored the whole partnership objectives.”* P28: KII_ Former Administrator _PNFP.

All these developments culminated in a decline in actual amounts allocated by the central government to the PNFP sector. Some interview narratives also indicated that some district-level governments diverted funds originally allocated for PNFPs to finance other priorities.

Several responses aimed at addressing the problems related to trust and probity. All PNFP bureaus embraced the use of government planning and information systems during this phase. The internal accreditation system created by the bureaus was strengthened to comply with MoH guidelines [10, 16, 26, 46, 48, 57]. PNFPs increased transparency and accountability by providing timely performance reports on outputs to district governments and the MoH; they also invested in tracking and reporting financial resource contributions from all sources (fees, grants and donations). Information sharing with government at district level and the MoH was institutionalized. All annual sector performance reports now reflect the contributions of the PNFP sector; these also document financial contributions that the PNFP sector receives from government, user fees and donations [61–64].

PNFPs have continued their ongoing advocacy to increase their share of the government health budget [26]. In response to ever-increasing costs of service provision, and reduced contributions from government sources, many PNFPs have increased charges for services. Raising charges remains a point of contention and mistrust, particularly between PNFPs and the communities they serve.

*“Because it [PHC grant] was not enough and yet not increasing, it created the temptation of wanting to increase the fees so as to enable us to bridge the financing gap in the hospital…but in so doing…in some cases this reduces the number of patients when the services became unaffordable and therefore inaccessible.”* P21: KII_ Administrator_ Facility_ District level_ Government.

Reduction in the volume of subsidized services have been reported because of reduced PHC grants to PNFPs. Many PNFPs have adopted vigorous resource mobilization efforts from alternative sources to sustain services. HIV programs for example have served as alternative sources of funds for PNFPs to compensate for the reduction of PHC grants from the government [65, 66]. Thanks to their autonomy, and their coverage of the rural poor, PNFPs have continued to attract donor funds to fill gaps left by reduced PHC grants.

## Discussion

The UHC agenda requires context-specific evidence on topics such as how quality health services can be extended to communities in need. In a pluralistic health system like Uganda’s, where the public and private sectors co-exist and each plays a critical role in service provision, policies to offset costs of or subsidize private service provision are important. This paper describes how GRCs, in the form of PHC grants, contributed to increasing service coverage by subsidizing PNFPs. Through a complex adaptive systems lens, this study demonstrates the adaptations made by each party in the government-PNFP relationship.

Over the period from 1997 to 2015, the relationship between government and PNFPs has undergone three major phases: 1) initiation, 2) increase and 3) decline in funding through PHC grants. This study mentions a number of adjustments made by the government and the UCMB network in particular to respond to changes in PHC grants while sustaining service provision, especially for the poor. The dynamics of this relationship provides lessons that can be applied to PNFPs more broadly. These will be vital in shaping policies to scale up GRCs to PNFPs and in addressing the challenges likely to be encountered during implementation.

The trend analysis revealed increases in key services in the UCMB network during the study period, despite the decreasing value of PHC grants. Explanatory factors for variations in PHC grants included the need to compensate for financial deficits at PNFP facilities, successful advocacy by PNFP managers, “good will” in the government facilitated by champions, the expanded government resource envelope, donor aid financing mechanisms and modalities, and changes to user fee policies. Increases in PHC grants led to several positive responses among PNFPs, including increased professionalization, improved management capacities. Additionally, trend analysis showed that reductions in PHC grants led to increases in user fees charged by PNFPs.

Since 1997, government policy has existed to provide subsidies to the PNFP sector; however, contributions from government remained low relative to the operational costs of the PNFPs [17, 26, 42, 61, 65]. At the peak of government subsidies to PNFPs, in the early 2000s, its contribution was estimated to cover about 35% of operational costs for lower-level facilities and between five and 10 % of costs among hospitals in the PNFP sector [16]. Meanwhile, the costs of providing care continue to escalate, driving PNFPs to charge increasing fees for services. Increased wages in the public sector workforce created pressure to increase private sector salaries as well; the introduction of high cost technologies have also added to the burden. PNFPs have passed on the cost burden to the communities they serve [67].

Over the period of this case study, PNFPs increased the volume of services delivered. This is attributable to the increased number of PNFP providers, decreased user fees and donor grants for HIV, TB and malaria services. Results-based financing has also become a popular way for donors to mitigate the operational costs incurred by PNFPs and reduce the cost burden on communities [68].

Other benefits were stimulated by PHC grants to PNFPs. These included expansion of their networks, improving compliance with standards, and increased efficiency. However, adherence to standards costs money, propelling a vicious cycle of cost and user fee increases for all services, except those with vertical funding, such as HIV and TB that had to be provided free [69].

### Lessons learned from the dynamics that influenced PHC grants

Changes in the “good will” of the government towards PNFPs was one major factor underlying fluctuations in PHC grants. Policy champions in government and advocacy by PNFP managers for more funding for PNFPs have been key in sustaining PHC grants over time. The current Public Private Partnership for Health policy [70] and continued engagement of PNFPs in HPAC [26] provide opportunities for further advocacy. However, PNFP providers need to engage further with strategic stakeholders beyond the MoH, such as the MoFPED and Parliament. Advocacy is also required at lower levels, such as with leaders at district and community levels. PNFP providers need to develop a common advocacy agenda and to generate and use evidence to demonstrate their contributions to health sector goals. As noted in the study, generating good data on service utilization levels and resource contribution trends are a necessary first step in dealing with the perception problem of PNFP providers being considered non-transparent. For instance, several responses have been triggered within the UCMB network in response to PHC grants. Improved compliance with the standards of practice has been reported over the years; a requirement for accessing PHC funding is meeting minimum requirements for accreditation. Between 2008 and 2015, UCMB and other PNFP bureaus have worked to ensure that their facilities meet the standards [57]. In addition, they have monitored changes in user fees and experimented with alternative fee structures, such as flat fees, to reduce cost uncertainty among the communities they serve [42]. PHC support reportedly led to more efficient use of resources**.** Over time, increasing client loads has reduced operating costs among some PNFP providers. This line of thinking suggests that because of the low client turnout during the period before the receipt of PHC grants, PNFP facilities were operating with excess capacity in terms of supplies, personnel and infrastructure, making operational costs high.

Donor support modalities, such as HIPC debt relief initiatives, played a critical role in boosting government resources in early 2000s [30]. Pro-poor aid programs can be designed to advance a more holistic approach to health systems [71]. However, off-budget support and less flexible aid such as that provided by GHIs may not contribute to improving the government’s relationship with PNFPs [3, 36, 37]. Evidence shows that the relationship can be disrupted when donors bypass the government to work directly with the private sector, which may or may not include PNFPs. SWAps improved coordination and alignment of donor support with national priorities [37], and should be strengthened.

### Study limitations

This study faced a number of limitations that may have affected its results. First, the long time-frame being discussed creates challenges of recall bias. To try to account for this, we triangulated sources and held the validation meetings to generate a collective memory of key events. Second, it was not possible to ascribe the contribution of PHC grants to the increase in coverage from trends in service outputs, particularly in the absence of population denominators. Changes in service outputs, for example, could have been due to several factors. The conceptual linkages constructed using CLDs sought to capture at least some of these influences. Third, the case study focused on UCMB; while instructive, this focus could limit the transferability of findings. We reached out to other medical bureau managers to get their perspectives on the findings. A larger study encompassing more PNFPs could more broadly identify factors in the relationship between bureaus and government. Further, a study with more regional variation would shed more light on equity concerns.

## Conclusions

Government resource contributions to the PNFP sub-sector in Uganda have gone through three main phases since 1997. Although the trend in GRCs to PNFPs declined in real terms towards the end of the study period, service outputs continued to increase. To sustain the growth in service outputs, UCMB and other PNFPs have increasingly resorted to mobilizing financial contribution from communities by increasing user fees. As the agenda for UHC takes center stage, contributions of resources from government to PNFPs should be revisited; financial allocations should be increased and strategic purchasing arrangements established that create explicit performance expectations for government funds.

PNFPs also need to develop strategies to limit operational costs, particularly as new and expensive technologies are introduced and wages for health workers increase. Deliberate and sustained engagement and advocacy by PNFPs at national and district levels are required to ensure that partnerships between government and PNFPs make meaningful and documented contributions to UHC goals. PNFPs can play a particularly important role in serving rural areas not reached by the public system. Policy and program design for UHC by government, development partners and GHIs alike should recognize and leverage the PNFP sector’s extensive network of infrastructure and human resources.
